# A Rare Case of Presumed Insulin-Like Growth Factor 2 (IGF-2)-Mediated Hypoglycemia

**DOI:** 10.7759/cureus.95913

**Published:** 2025-11-01

**Authors:** Kunal Gupta, Adlyne Reena Asirvatham, Sandhya Sundaram, Pushpa Machineni, Sai Namratha Gogineni, Shriraam Mahadevan

**Affiliations:** 1 Endocrinology, Diabetes and Metabolism, Sri Ramachandra Institute of Higher Education and Research, Chennai, IND; 2 Endocrinology, Sri Ramachandra Institute of Higher Education and Research, Chennai, IND; 3 Pathology, Sri Ramachandra Institute of Higher Education and Research, Chennai, IND

**Keywords:** gastrointestinal stromal tumour, hypoglycemia, igf-2, imatinib, non-diabetic hypoglycemia, non-islet cell tumour hypoglycemia

## Abstract

Non-diabetic hypoglycemia is an uncommon yet potentially life-threatening medical disorder in adults. An atypical cause is non-islet cell tumor hypoglycemia (NICTH), frequently mediated by insulin-like growth factor 2 (IGF-2) released by mesenchymal tumors, including gastrointestinal stromal tumors (GISTs).

We discuss the case of a 75-year-old male who exhibited recurring bouts of altered sensorium, disorientation, and excessive sweating during a three-month period, all of which resolved upon food consumption. Preliminary assessments indicated hypoglycemia (capillary blood glucose 35-40 mg/dL) without any prior history of diabetes, insulin, or sulfonylurea administration. A monitored 72-hour fasting test revealed hypoglycemia (capillary blood glucose (CBG) 42 mg/dL), suppressed insulin (<0.20 µIU/mL), and C-peptide (0.12 ng/mL), thereby excluding insulinoma and factitious hypoglycemia. Imaging identified an intra-abdominal mass, and histopathological analysis corroborated the diagnosis of gastrointestinal stromal tumor (GIST). The clinical presentation was suggestive of a presumed IGF-2-mediated NICTH. In light of the lack of metastatic dissemination and the patient's advanced age, targeted therapy with imatinib was commenced, leading to the elimination of hypoglycemia episodes.

This case underscores the necessity of incorporating NICTH into the differential diagnosis of hypoglycemia in non-diabetic individuals, particularly when a tumor is present. Timely identification and focused intervention of the underlying neoplasm can result in positive outcomes.

## Introduction

The diagnosis of hypoglycemia in non-diabetic persons is rare [1]. A variety of etiologies, such as adrenal insufficiency, sepsis, hepatic dysfunction, renal failure, malnutrition, alcohol-related disorders, malignancies, including islet cell tumors, pancreatic hypertrophy from bariatric surgery, and pharmacological agents, may lead to hypoglycemia in both non-diabetic and diabetic patients [2]. A thorough history and examination can help in the diagnosis. Identifying the presence of Whipple’s triad is crucial for distinguishing between pathological and physiological hypoglycemia [3]. Identifying and addressing the underlying cause of hypoglycemia is essential.

Non-islet cell tumor hypoglycemia (NICTH) is one of the rarest types of hypoglycemia encountered in clinical practice. The predominant cases of NICTH are mediated by insulin-like growth factor 2 (IGF-2), a bioactive polypeptide that is rarely overexpressed by tumors of mesenchymal or epithelial origin. IGF-2 demonstrates insulin-mimetic properties and induces hypoglycemia through its interaction with IGF receptors and insulin receptors.

NICTH has been linked to carcinomas such as breast, cervical, lung, gastric, hepatocellular, colon, and pancreatic, in addition to different vascular and mesenchymal cancers [4,5]. Excessive synthesis by tumor cells can result in heightened glucose uptake by tissues, augmented glucose use, and diminished glucose production by the liver, culminating in hypoglycemia and symptoms, including disorientation, weakness, dizziness, and loss of consciousness [6]. Individuals with IGF-2-mediated hypoglycemia exhibit fasting hypoglycemia marked by diminished endogenous insulin, ketones, growth hormone, and IGF-1 levels. The primary treatment for hypoglycemia is predominantly supportive, but the excision of the underlying tumor is curative in the majority of instances [7].

Here, we present a rare case of a presumed IGF-2-mediated non-diabetic hypoglycemia in an elderly male.

## Case presentation

A 75-year-old male exhibited recurrent bouts of altered sensorium during a three-month period. These episodes were marked by disorientation, irrelevant speech, atypical behavior, such as concealing oneself beneath the bed, and excessive perspiration. The symptoms abated swiftly following consumption of meals. No history of loss of consciousness or seizure activity was recorded during these incidents. He is a known case of systemic hypertension on Telmisartan + chlorothiazide therapy. An initial assessment at a nearby healthcare institution indicated hyponatremia (Na⁺ 121 mEq/L), ascribed to thiazide-induced sodium depletion. Management commenced for electrolyte correction, and the patient was instructed to monitor capillary blood glucose (CBG) levels at home. Notwithstanding these interventions, the patient persisted in experiencing analogous episodes, during which CBG levels fluctuated between 35 and 40 mg/dL. Due to the recurrence of these hypoglycemia incidents, he was referred for additional assessment.

No prior history of diabetic mellitus, corticosteroid treatment, or usage of traditional or alternative therapies was documented. Furthermore, there was no personal or familial history indicative of endocrine or autoimmune problems. The patient reported no prior or current usage of insulin or sulfonylurea, excluding the likelihood of iatrogenic hypoglycemia.

At the time of examination, the patient was alert, oriented, and hemodynamically stable, with a pulse rate of 88 beats per minute and a blood pressure of 120/70 mmHg. The physical examination revealed no significant findings, except for a firm, non-tender epigastric mass extending to the umbilical region, observed during abdominal palpation.

Investigations

Standard laboratory assessments were conducted, indicating a glycated hemoglobin (HbA1c) of 5.2%, a random blood glucose level of 102 mg/dL, serum creatinine of 0.7 mg/dL, and normal liver function tests. These findings ruled out diabetes mellitus as a cause, focusing the diagnostic focus toward non-diabetic hypoglycemia.

The patient was admitted for a supervised 72-hour extended fasting test, with capillary blood glucose monitored every two hours. After four hours of fasting, he exhibited symptomatic hypoglycemia, with a capillary blood glucose level of 42 mg/dL, confirmed by a random blood glucose measurement of 50 mg/dL. A critical sample obtained during hypoglycemia indicated suppressed insulin (<0.20 µIU/mL) and C-peptide (0.12 ng/mL) levels, thereby effectively excluding insulinoma and factitious hypoglycemia. Serum cortisol was 6.15 µg/dL, which we attribute to hypoglycemia-induced adrenal insufficiency.

**Figure 1:**
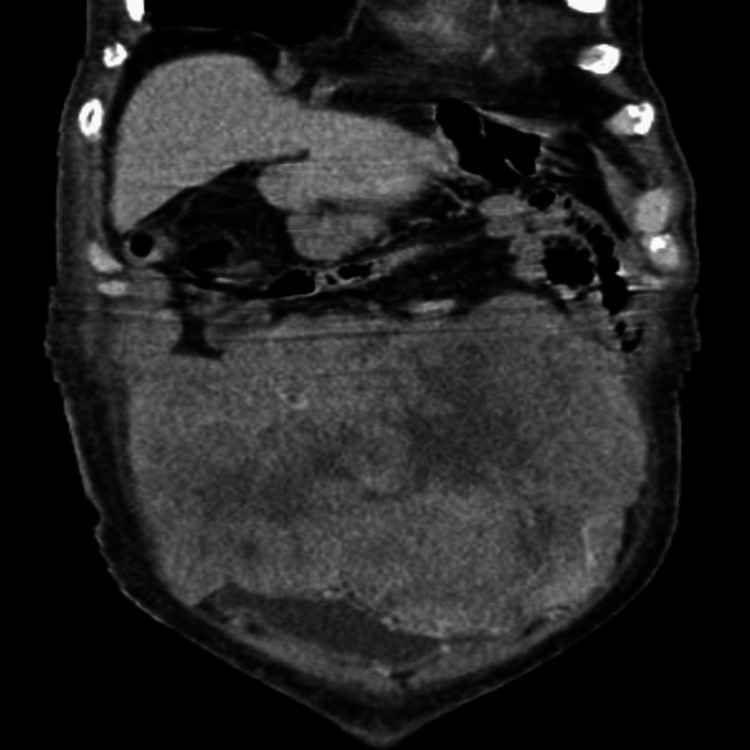
CT abdomen showing a large abdominopelvic mass lesion

**Figure 2:**
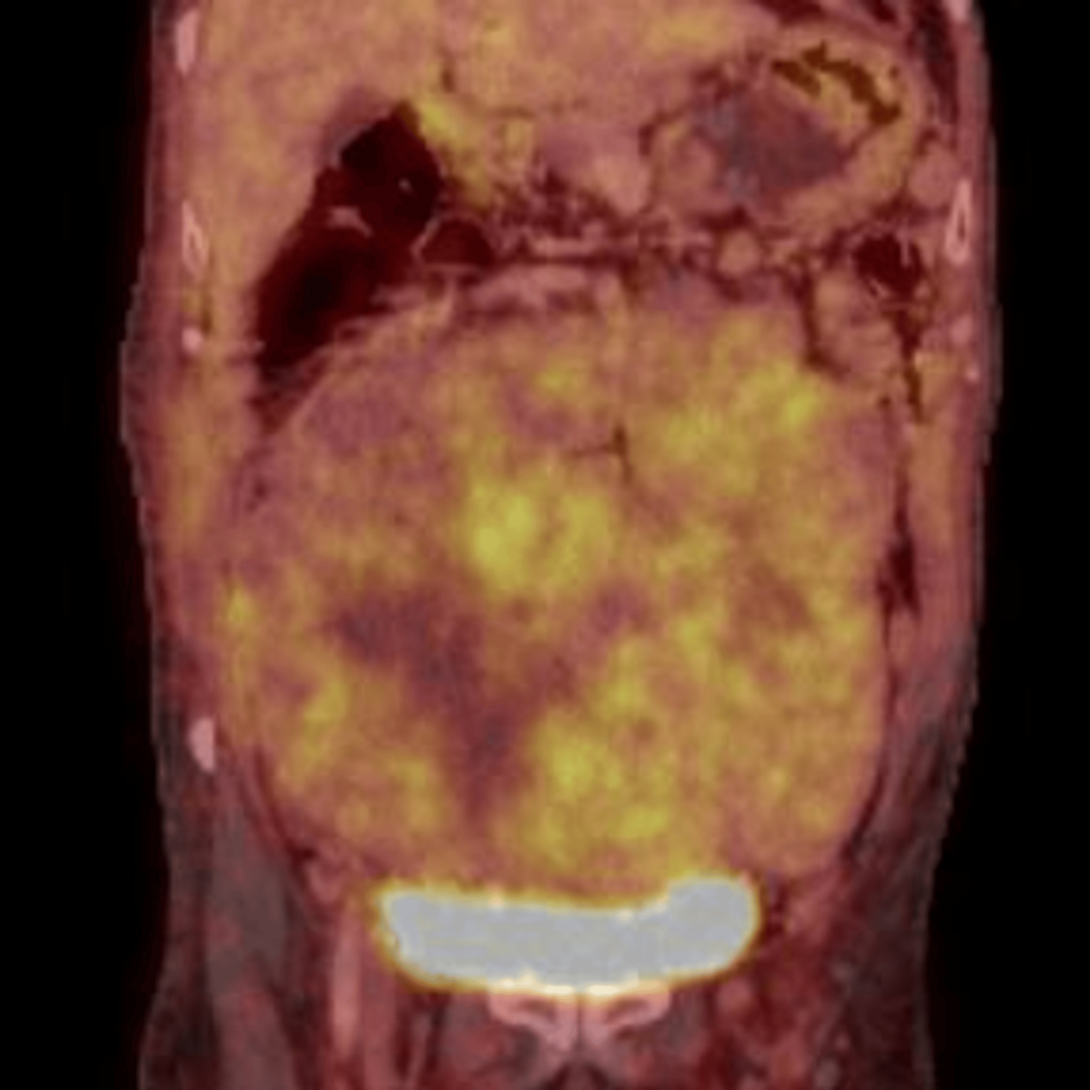
F-18 FDG PET-CT: abdominopelvic lesion showing avid FDG uptake F-18 FDG: F18 fludeoxyglucose; PET: positron emission tomography; CT: computed tomography

The diminished insulin and C-peptide levels, together with the patient's medication history and the lack of pertinent autoimmunity, ruled out insulinoma, factitious hypoglycemia from exogenous insulin or sulfonylureas, autoimmune hypoglycemia, and adrenal insufficiency. The moderate cortisol suppression was inconsistent with a typical Addisonian crisis, hence reducing the probability of adrenal disease. In light of an abdominal mass and the elimination of other etiologies, radiographic imaging was conducted. An abdominal ultrasound identified a heterogeneous mass comprising both solid and cystic elements, measuring approximately 17 × 16 × 6 cm. A contrast-enhanced computed tomography (CT)-guided biopsy was conducted (Figure 1), and histological examination validated the diagnosis of a gastrointestinal stromal tumor. A second whole-body positron emission tomography (PET)-CT scan revealed a fluorodeoxyglucose (FDG)-avid lesion situated in the mesentery, with no indications of distant metastases (Figure 2).

The existence of a tumor, coupled with diminished insulin and C-peptide levels amid recurrent fasting hypoglycemia, heightened the suspicion for NICTH. This disease is characterized by the overproduction of IGF-2 or its high-molecular-weight precursor, "big IGF-2," which emulates insulin activity, resulting in hypoglycemia.

Management

A presumptive diagnosis of IGF-2-mediated non-diabetic hypoglycemia resulting from GIST (NICTH) was made. Given the patient's advanced age and lack of metastatic disease, a non-surgical treatment strategy was implemented. He was commenced on imatinib, a tyrosine kinase inhibitor recognized for its effectiveness in treating GISTs. Upon commencement of targeted therapy, within three weeks, the patient demonstrated substantial clinical enhancement with total remission of hypoglycemia episodes.

Upon follow-up, the patient is clinically stable with no further episodes of hypoglycemia. He is undergoing routine outpatient surveillance, with intermittent imaging to evaluate tumor response and determine the necessity for future action. The biochemical investigations are listed in Table 1.

**Table 1:** Biochemical investigations AST: aspartate aminotransferase; SGOT: serum glutamic-oxaloacetic transaminase; ALT: alanine aminotransferase; SGPT: serum glutamate pyruvate transaminase

Parameter	Patient Value	Reference Range	Interpretation
Capillary Blood Glucose (CBG) during episode	35–42 mg/dL	70–110 mg/dL (fasting)	Markedly low – hypoglycemia
Random Blood Glucose	50 mg/dL	70–140 mg/dL	Low
HbA1c	5.20%	4.0–5.6 %	Normal – excludes diabetes
Serum Insulin (during hypoglycemia)	< 0.20 µIU/mL	2.6–24.9 µIU/mL (fasting)	Suppressed
C-peptide (during hypoglycemia)	0.12 ng/mL	0.9–7.1 ng/mL	Suppressed
Serum Cortisol	6.15 µg/dL	6–23 µg/dL (morning)	Low-normal (stress-related suppression likely)
Serum Sodium (Na⁺)	121 mEq/L	135–145 mEq/L	Hyponatremia (thiazide-induced)
Serum Creatinine	0.7 mg/dL	0.6–1.3 mg/dL	Normal renal function
AST (SGOT)	32 U/L	≤ 40 U/L	Normal
ALT (SGPT)	36 U/L	≤ 41 U/L	Normal
Total Bilirubin	0.9 mg/dL	≤ 1.2 mg/dL	Normal
Direct Bilirubin	0.7 mg/dL	≤ 0.3 mg/dL	Mildly elevated
Indirect Bilirubin	0.2 mg/dL	0.2–0.9 mg/dL	Normal

## Discussion

The presumed mechanism of hypoglycemia in this case involves excessive tumor synthesis of mature IGF-2 and the immature “large” pro-IGF-2, which interact with IGF and insulin receptors. This results in hypoglycemia through multiple pathways, including enhanced peripheral glucose uptake, diminished lipolysis, reduced free fatty acids, decreased hepatic gluconeogenesis, impaired glycogenolysis, and inhibition of anterior pituitary growth hormone secretion. Patients consequently exhibit fasting hypoglycemia, as demonstrated by Whipple’s triad [1].

In our case, the 75-year-old patient experienced recurrent episodes of impaired sensorium, disorientation, and autonomic symptoms, including profuse sweating, which were routinely alleviated by food intake, indicating hypoglycemic events. The absence of diabetes and low capillary blood glucose during symptomatic episodes prompted a thorough evaluation to determine the underlying cause [2].

Preliminary assessment excluded common causes of hypoglycemia, including exogenous insulin or sulfonylurea administration, insulinoma, autoimmune hypoglycemia, adrenal insufficiency, and hepatic or renal dysfunction. The 72-hour monitored fasting test was particularly crucial, as it simulated hypoglycemic episodes under controlled conditions and allowed for the collection of critical samples. Reduced insulin and C-peptide levels during hypoglycemia effectively ruled out insulinoma and exogenous insulin use, indicating a non-insulin-mediated mechanism [3].

The identification of an epigastric mass, alongside imaging and histological confirmation of a gastrointestinal stromal tumor (GIST), raised suspicion for NICTH. NICTH is a paraneoplastic condition caused by the excessive production of IGF-2 or its high-molecular-weight precursor, “big IGF-2.” These molecules mimic insulin’s effects by increasing peripheral glucose uptake and inhibiting hepatic glucose production, leading to sustained hypoglycemia. Unlike insulin-mediated hypoglycemia, IGF-2-induced hypoglycemia is characterized by low insulin, low C-peptide, low β-hydroxybutyrate, and variable cortisol levels [4,5].

GISTs are rare mesenchymal tumors, typically arising in the stomach or small intestine, and can, in exceptional cases, secrete IGF-2 and cause hypoglycemia via the NICTH pathway [6,7]. Hypoglycemia may precede or coincide with tumor detection. Imaging studies, such as PET-CT, are vital to define the extent of disease and exclude metastases, thereby guiding individualized management strategies [8].

Management of hypoglycemia initially involves oral or parenteral dextrose administration. However, IGF-2-mediated hypoglycemia often recurs despite optimal nutritional intake. In localized disease, surgical resection is curative and typically normalizes the IGF-2/IGF-1 ratio [9,10]. For metastatic or unresectable tumors, debulking, systemic chemotherapy, radiotherapy, or vascular embolization may help alleviate hypoglycemia [10].

Pharmacologic interventions are also effective. Glucocorticoids inhibit pro-IGF-2 synthesis, enhance gluconeogenesis, promote lipolysis, and reduce peripheral glucose uptake [11,12]. Common agents include prednisone, prednisolone, methylprednisolone, and dexamethasone. High-dose subcutaneous recombinant growth hormone (rGH) therapy may be beneficial but carries risks such as edema, skin tags, and exacerbation of arthritic pain [12].

In our patient, NICTH management targeted the underlying tumor. Although surgical resection is definitive, it may not be feasible in elderly patients or those with unresectable disease. The patient was started on imatinib, a tyrosine kinase inhibitor effective against KIT-positive GISTs, which led to the resolution of hypoglycemia and clinical stabilization [8].

Several case reports and series have documented similar NICTH manifestations arising from hepatocellular carcinoma, fibrosarcoma, and GISTs. These reports emphasize the need for heightened clinical suspicion, particularly when hypoglycemia occurs with low insulin and C-peptide levels in non-diabetic patients with confirmed or suspected malignancies [13]. Pharmacologic interventions are also effective. Glucocorticoids inhibit pro-IGF-2 synthesis, enhance gluconeogenesis, promote lipolysis, and reduce peripheral glucose uptake [12,13]. Common agents include prednisone, prednisolone, methylprednisolone, and dexamethasone. High-dose subcutaneous rGH therapy may be beneficial but carries risks such as edema, skin tags, and exacerbation of arthritic pain [12]. Newer pharmacologic options, such as pasireotide, have also been reported to control tumor-induced hypoglycemia in both insulinoma and non-islet cell tumor hypoglycemia [14].

## Conclusions

This case highlights the necessity of evaluating non-islet cell tumor hypoglycemia (NICTH) in the differential diagnosis of recurring, unexplained hypoglycemia, especially in non-diabetic elderly individuals. Although insulinoma is typically the primary suspicion, diminished insulin and C-peptide levels during hypoglycemia necessitate investigation for other etiologies, including insulin-like growth factor 2 (IGF-2)-secreting neoplasms like gastrointestinal stromal tumors (GISTs). Prompt diagnosis and focused treatment, exemplified by imatinib in this patient, can lead to substantial clinical enhancement and the alleviation of hypoglycemia incidents. Clinicians must uphold a heightened level of suspicion for NICTH in patients exhibiting atypical hypoglycemia, particularly when accompanied by a palpable mass or radiological indications of a malignancy.
